# Predicting condom use in adolescents: a test of three socio-cognitive models using a structural equation modeling approach

**DOI:** 10.1186/s12889-016-2702-0

**Published:** 2016-01-14

**Authors:** José P. Espada, Alexandra Morales, Alejandro Guillén-Riquelme, Rafael Ballester, Mireia Orgilés

**Affiliations:** 1 Department of Health Psychology, Miguel Hernández University, Av. de la Universidad, s/n, 03202 Elche, Alicante, Spain; 2 Department of Personality, Evaluation and Psychological Treatment, University of Granada, Campus Universitario de Cartuja, 18011 Granada, Spain; 3 Department of Basic and Clinical Psychology and Psychobiology, Jaume I University of Castellón, Avda. Sos Baynat, s/n, 12071 Castellón de la Plana, Castellón, Spain

**Keywords:** Theoretical models, Adolescents, Condom use, Prevention, HIV, STIs, Unplanned pregnancy

## Abstract

**Background:**

The theory of planned behavior (TPB), socio-cognitive model (SCM), and information-motivation-behavioral skills (IMB) model are effective in predicting condom use. However, the adequacy of these three theoretical models in predicting the frequency of condom use (FCU) among young people has not been compared. This cross-sectional study tested the applicability and suitability of these three models in predicting the FCU, and analyzed the relationships among the postulated constructs.

**Methods:**

Sexually experienced adolescents (*n* = 410) aged 13–18 completed a survey assessing the TPB, SCM, and IMB model constructs. Participants were students recruited from 18 high schools, randomly selected from the north, south, east, and southeast of Spain. A structural equation modelling (SEM) analysis was applied to test TPB, SCM and IBM and constructs relationships of each model using R.

**Results:**

The results of SEM demonstrated that behavioral skills predict behavior via motivation as hypothesized by the IMB model, but not directly via knowledge about condom use and sexually transmitted infections (STIs). Cognitive factors, such knowledge about condom use and STIs as well as condom use self-efficacy, directly predicted the FCU when modeled as per the SCM. According to the TPB, condom use intention was the best predictor of the FCU, and condom use intention was predicted by attitudes toward condom use and subjective norms related to condom use, but perceived control was not directly or indirectly related to the FCU. Based on the data, the TPB becomes the best-fit model for predicting the FCU among young people compared to the SCM and IMB model.

**Conclusions:**

From a statistical perspective, the TPB seems to be the most suitable model for predicting the FCU among young people compared to the other models. Overall, key direct predictors of the FCU in adolescents included condom use intention, behavioral skills and cognitive factors, such as STIs knowledge and condom use self-efficacy. The next step should be to test integrative models that include personal, contextual, environmental, and social factors.

**Electronic supplementary material:**

The online version of this article (doi:10.1186/s12889-016-2702-0) contains supplementary material, which is available to authorized users.

## Background

Young people (aged 15–24 years) have contributed to a recently reported reduction in the incidence of sexually transmitted infections (STIs); this is perhaps due to their greater awareness of STIs, including human immunodeficiency virus (HIV), and their increased engagement in safer sexual behaviors [1]. Despite these data, adolescents are still considered a high-risk population for engaging in risky sexual behavior worldwide [1, 2]. Research on condom use predictors among adolescents has attracted interest from preventers and clinicians in order to reduce risky sexual behavior in this population.

The interventions with greater empirical evidence regarding the prevention of contracting STIs are based on socio-cognitive theories [3]. These are social-cognitive models that describe the key variables and determinant interrelationships in predicting health behaviors [4]. Behavior modification through preventive actions for the social and cognitive factors attempts to engage individuals in healthy behaviors, such as consistent condom use. Among the most widely used theoretical models in the field of HIV/AIDS are the theory of planned behavior (TPB) [5–7], socio-cognitive model (SCM) [8–10], and information-motivation-behavioral skills (IMB) model [11–13].

According to the TPB, intention is the best predictor of behavior, and this is determined by attitudes, normative beliefs toward such behaviors, and perceived control. The attitudinal component is defined as the set of beliefs regarding the behavior’s value and its consequences. Attitude toward condom use is more favorable when negative consequences of not using a condom are valued (STIs or unwanted pregnancy) and the benefits of its use (condom use as an exciting element) were valued as being positive [4]. The normative component represents the perception of what the reference groups do and the personal motivation to act in accordance with the reference group’s expectations. This construct encompasses individuals’ perceptions of how the reference group behaves (descriptive norms), and what is expected of the individual in relation to such behavior (injunctive norms). An individual will use a condom during sex if they perceive that the peer group does (i.e., acting according to group pressure). Perceived control refers to the perceived ability to perform a behavior depending on resources and personal limitations. Ajzen [5] suggested that perceived control may also directly predict behavior when the behavior is complex or not under volitional control.

Meta-analytic and review studies provide extensive empirical support of the TPB in predicting condom use and other health behaviors across different populations, such as adolescents and college students [14–20]. In a meta-analytic review of 185 studies, Armitage and Conner [19] observed that the TPB accounted for 39 and 27 % of the variance in intention and behavior, respectively. Godin and Kok [14] found similar results in a review of 56 studies testing the applicability of the TPB to health-related behaviors (41 and 34 % explained variance for intention and behavior, respectively). More recently, McEachan and colleagues [18] concluded that the TPB shows a poorer explanation regarding safer sex (variance explained from 13.8 to 15.3 %) compared to other health behaviors, such as physical activity (24) and diet behaviors (21 %). Generally, perceived norms show a weaker contribution to predict intention compared to attitude and perceived control [14, 15, 18, 19]. Albarracín et al. [15] meta-analyzed 96 studies predominantly conducted in Europe and United States, and concluded that attitude is the best predictor of condom use intention (*r* = 0.58; β = 0.47) followed by perceived control (*r* = 0.45, β = 0.20), and perceived norms (*r* = 0.39; β = 0.20). However, perceived norms have shown to be more predictive of the behavior in adolescents compared to adults [18]. More evidence about the contribution of the intention’s predictors is needed, especially in adolescent samples.

The SCM [8–10] is the most commonly used theory in HIV prevention for young people worldwide [21]. From the social-cognitive perspective, behavior is determined by the reciprocal, dynamic, and continuous interaction between the personal (cognitive component), behavior (behavioral component), and environment (social component) [22]. The cognitive component refers to the individual’s confidence in their ability to perform a task or achieve a particular goal (i.e., high self-efficacy to exercise over one’s sexual behavior). The behavioral component encompasses individual consequences of performing a behavior and influences the chances of the learner behaving correctly by practice (i.e., skills necessary to enable an individual physically capable of performing a behavior to obtain the desired outcomes). An application to sexual health could be the valuable skills in managing interpersonal situations and protecting themselves against STIs and unplanned pregnancies [9]. The social component involves aspects of the environment that improve the individual’s ability to successfully undertake specific behaviors. It refers to vicarious learning and social consequences of behavior. People develop behavioral competences by learning directly from their actions or through social modelling (observing how other people belonging to the same physical and social environments behave and/or consequences of their behavior) [23]. Adolescents whose friends used condoms would be more likely themselves to use condoms. The SCM has served as a theoretical framework to evaluate the efficacy of HIV-risk reduction interventions and to predict condom use in high-risk populations belonging to low-income cities, such as adolescents, college students, and drug users [9, 24–27].

From a critical review of the theoretical models, Fisher and Fisher [11–13, 28, 29] developed the IMB model, which included relevant social and psychological constructs [7, 30]. Although the IMB model has a broad application in health promotion, it was initially developed to reduce sexual risk behaviors and HIV infection [12]. The IMB model has demonstrated its appropriateness as a framework for predicting sexual risk, primarily with the adult population, although also with adolescents [12, 29, 31, 32]. Most of these studies were conducted in low- and middle-income countries [33–35]. From this approach, information, motivation, and behavioral skills are determinants of HIV-prevention behavior. The information component includes what HIV is, the risk involved in having unprotected sex, and protection methods. Knowledge is a prerequisite in this explanatory model of sexual behavior [28]. The motivational component refers to the motivation to engage in safe sex and avoid risky behaviors. This includes attitudes toward condom use, perception of social support, and intentions to engage in safe sex. The behavioral skills component comprises the individual’s ability to carry a behavior, and the perceived self-efficacy in the practice of the behavior [9, 29, 30]. Perceived self-efficacy refers to the perception of control over motivation, thought processes, emotional states, and behavior [9].

A review of the intervention strategies based on the IMB model for health behavior change concluded that it is applicable for promoting healthy behaviors in chronic patients (e.g., heart disease self-care, diabetes, etc.) and for interventions focusing on reducing risk by promotion of consistent condom use [36]. The IMB model accounted for the explained condom use variance of 19 % in students from Cape Town, South Africa [37]. In a cross-sectional study involving 3,183 college students aged 16 to 25 years in China [38], consistent condom use was significantly predicted by behavioral skills (β = 0.75), although it was not directly related to information and motivation.

These socio-cognitive models—TPB, SCM, and IMB model—specify variables that can determine whether someone will engage in a healthy behavior, such as using condoms during sex. Some of these variables are common in the TPB, SCM, and IMB model; however, their conceptualization may differ across the models [39]. Each of these theories assumes the relevance of having a positive attitude toward the behavior and peer influence to use condoms during sex. Perceived norms emphasize people’s perception of social normative pressures or other’s beliefs that they should or should not perform a particular behavior in the TPB. However, in the SCM, it refers to vicarious learning and social consequences of behavior. Self-efficacy is a predictor of intention in the TPB, is part of the behavioral factor in the SCM, and is key in the behavioral skills in the IMB model. Self-efficacy refers to the belief in one’s ability to succeed in specific situations. Compared to the TPB and SCM, the IMB model does not emphasize confidence that the action can be undertaken in adverse conditions [8]. Intention is the best predictor of condom use in the TPB, while it is part of the motivational component in the IMB model. Compared to the TPB, the informational component is present in the IMB model and cognitive factors of the SCM, noting the need to have sufficient knowledge about methods of protection to use condoms during sex.

Extensive empirical evidence supports the adequacy of the TPB, SCM, and IMB model in predicting healthy behaviors in general [7, 9, 29], particularly condom use in adolescents and college students [9, 12, 25, 35, 40–42]. However, no studies thus far have compared the suitability of these three models for condom use prediction in adolescents. According to Noar and Zimmerman [43], several reasons justify the comparison of these health behavior theories. First, this allows us to identify the theory most accurate to predicting health behaviors. Second, it examines the similarity and difference across models. Third, it provides relevant information about the relationships among theoretical constructs, and identifies which ones are more relevant to predict the behavior and must be included in health promotion interventions. Although the empirical comparison of health behavior theories is essential in research to move forward, studies with this aim are rarely found in the scientific literature [39, 43].

The present study aims to investigate which socio-cognitive models—TPB, SCM, or IMB—best predict the frequency of condom use (FCU) in adolescents [1, 2]. Determining the weight of each variable in predicting the FCU is of great value when designing interventions that promote consistent condom use. This study’s first objective was to determine the applicability and suitability of these theories of health behavior in predicting the FCU among Spanish adolescents. The applicability and suitability of each model was determinate by the model fit indices to predict the primary outcome. A good-fitting model (in terms of the statistical indices of CFI, TLI, and RMSEA) indicated a better applicability and suitability of the model for its purpose. The second objective was to analyze the relationships among the constructs postulated by these models and their contribution to the prediction of the FCU. Based on previous research, we hypothesized that three models will be suitable to predict FCU among Spanish adolescents. According to the theoretical models, the variables involved in predicting condom use will present a high and significant relationship.

## Methods

### Participants

A total of 1,562 adolescents aged 13 to 18 participated from four autonomous communities in the north, south, east, and southeast of Spain. Participants who had experienced vaginal intercourse (*n* = 410; 39 % were female) were selected for the study since FCU was the main outcome. The mean age of this subsample was 15.54 years (*SD* = 1.07) for women and 15.58 (*SD* = 1.06) for men. According to the Family Affluence Scale, 3.2 % of the participants were of low socioeconomic status, 54.9 of medium status, and 41.9 % of high status [44].

### Procedure

This cross-sectional study is part of a wider project aiming to evaluate the effectiveness of two programs for sexual health promotion in Spanish adolescents. Baseline data – collected from January to March 2012 - were used to avoid contamination of the answers by the program’s content. This project brings together five Spanish universities located in four geographical areas (north, south, central, and southeastern) of Spain. The ethics committee from the Miguel Hernandez University approved the present study (DPS-JPE-001–10). Eighteen of twenty-one public schools invited to participate were enrolled in the study. The three non-participating schools had incompatible activities with this project. The project coordinator randomly selected three schools from each province (Alicante, Asturias, Castellón, Granada, and Murcia). The sample size was determined by available funding to implement the trial (300 participants per province). Three additional schools were randomized in order to add to the previously fixed sample size. In each research center, a member of the team was responsible for visiting the schools in their area to request the participation of these schools in the study. Students’ parents were informed about the study and received a written informed consent by a member of the team at the parents-teacher meeting, celebrated at the beginning of the course. Parents’ doubts were discussed and solved at that time. Additionally, a phone number and an email address were included in the consent so that parents could request more information and ask questions during the process. Students enrolled in Grades 9 and 10 (in 2012) and those who had provided written informed consent signed by their parents were eligible to participate in the study. The rate of acceptance for participating in the study was high (97 %). Eligible participants were informed about the study objective and the confidentiality of the data was ensured. The assessment was undertaken in groups of approximately 30 students at high schools. Whenever possible, they were undertaken online using *Google Forms*; however, for the centers that lacked computing resources, the questionnaires were provided in paper form (Additional file 1). A member of the research team remained in the class during the entire assessment to answer questions if participants had any and they restarted the software after each assessment. The approximate duration of each assessment was 50 min. No participant withdrew from the study once they initiated the survey. No incentives were provided to the participants who answered the survey.

### Measures

The theoretical models’ variables were evaluated using direct questions and instruments validated in previous studies with adolescents.

#### Attitude towards condom use

This was evaluated using four items with four response alternatives from 1 (*strongly disagree*) to 4 (*strongly agree*). An example of an item is “Using a condom during sex consistently is good to prevent STI transmission and unplanned pregnancies”. In this sample, the internal consistency of this subscale was acceptable (α = 0.74).

#### Condom use self-efficacy

We applied the condom use self-efficacy subscale from the HIV Attitudes Scale (HIV-AS) [45]. The subscale consists of three items with four response alternatives from 1 (*strongly disagree*) to 4 (*strongly agree*). An example of an item is “If my partner would want to have sex without a condom, I would try to convince her/him to use it”. In this sample, the internal consistency of this subscale was acceptable (α = 0.76).

#### Perception of condom availability

This was evaluated using items regarding the perception of how easy is to obtain condoms: (1) “Do you think that it is easy to get condoms?”.

#### Intention to use condoms

This was assessed using one item rated on a 5-point Likert scale, from 1 (*not sure*) to 5 (*sure*): “I will use a condom if I have a sexual relationship”.

#### Knowledge about HIV and other STIs

The *Escala de Conocimiento sobre ITS* (ECI) was the instrument used to measure the participants’ knowledge about HIV and STIs [46]. The scale consists of 24 items with true/false answers. The following factors were evaluated, (1) general knowledge about HIV, (2) condom use to prevent HIV, (3) routes of transmission of HIV, and (4) knowledge about other STIs. The total score range is 0–24. The internal consistency is acceptable for Spanish adolescents (α = 0.88).

#### Social skills

The Matson Evaluation of Social Skills with Youngsters (MESSY) was used [47]; this scale has been validated for a Spanish adolescent population [48]. The questionnaire consists of 62 items rated on a 5-point Likert scale, ranging from 1 (*it does not describe me at all*) to 5 (*it describes me a lot*). It contains four factors: (1) Aggressive/Antisocial Behavior, (2) Social Skills/Assertiveness, (3) Arrogance/Vanity, and (4) Loneliness/Social Anxiety. Higher scores on the Social Skills/Assertiveness subscale indicate higher assertiveness; conversely, higher scores on the rest of the scales indicate lower social adjustment. The Spanish version of this questionnaire presents adequate internal consistency (α = 0.88) and convergent validity [48].

#### Subjective norms

This was evaluated using two items regarding the perception of condom use among peers: (1) “Do you think that your same-age friends use condoms in their sexual relationships?” and (2) “How often do you think your same-age friends use a condom in their sexual relationships?”.

#### Risk perception

The perception of sexual risk was evaluated using a 3-factor scale related to the perceived risk of HIV (2 items), other STIs HIV (2 items), or an unplanned pregnancy HIV (2 items). For example: ‘How much risk is there of transmitting HIV by engaging in vaginal sex without a condom?’ and ‘How much risk is there of transmitting HIV by engaging in oral sex without a condom?’ The average maximum total of factor is 8, on a scale from 1 (no at all) to 4 (a lot). The internal consistency of the instrument was high (0.87).

#### Sexual behavior

Participants responded to the following questions: (1) sexual experience (“Have you ever had vaginal sex?”) with a dichotomous response (yes/no)—adolescents who stated that they had never had vaginal sex were excluded from the analysis, (2) age at the first instance of sexual intercourse, (3) sexual orientation (*heterosexual/bisexual/homosexual*), (4) condom use (“What percentage of times do you use a condom in your sexual relationships?”) with a response range from 0 % (never) to 100 % of the times (always), and (5) number of sexual partners in the past six months.

The questionnaire included socio-demographic (age and gender), family structure (living with married parents, divorced or separated parents, unmarried couples, single parents, or orphans), and family income [44] variables.

### Data analysis

Structural equation models were used to calculate the fit of the data to the theoretical models (The availability of data must be personally requested to the corresponding author at jpespada@umh.es). The robust Weighted Least Squares Mean and Variance (WLSMV) adjusted method was selected as it is suitable for use with categorical measures, and quantitative data as well [49]. The indices used for the comparison of the different models were the Comparative Fit Index (CFI), Tucker-Lewis Index (TLI), and Root Mean Square Error of Approximation (RMSEA).

According to Vandenberg and Lance [50], an acceptable model fit was determined by the values greater than 0.95 for an excellent fit. The RMSEA represents a good fit when it is equal to or less than 0.06, and 0.08 is the upper limit for a good fit. After the first adjustment of the models, changes recommended by the modification indices were included when they were higher than 10 and the relationships were consistent with the theory. We only included correlations between factors or items of the same construct to avoid possible covariance. Only cases without omissions were included in the analysis (*n* = 410). Because of the complexity of the analysis and sample size, variables as sexual orientation, age, age of first sexual experience were not controlled during the analysis. Simple descriptive statistics were employed to describe the sample of the study. The R package, Lavaan, was used to perform the analysis [51].

## Results

### Sample

The socio-demographic characteristics and sexual behaviors of participating students are described in Table 1. The group’s mean age was 15.56 (*SD* = 1.06) and 71.4 % were living with married parents, although 26.5 % had separated or divorced parents.

**Table 1:** Sociodemographic characteristics and self-reported behaviors of participating students

Characteristics	Total (*n* = 410)
No. (%) male	248 (60.5)
Mean age (*SD*), years	15.56 (1.06)
No. (%) Family structure	
Married parents	269 (71.4)
Divorced parents	100 (26.5)
Single parents	8 (2.1)
No. (%) Family income	
Low	19 (4.6)
Middle	231 (56.4)
High	160 (39)
No. (%) Sexual orientation	
Heterosexual	390 (96.5)
Bisexual	9 (2.2)
Gay/homosexual	5 (1.2)
Mean age (*SD*) first vaginal penetrative sex, years	14.57 (1.29)
Percentage of condom use (0–100) (*SD*)	85.47 (22.29)
No. (%) Consistent condom use	178 (51)
Mean (*SD*) Number of sexual partners in the past 6 months	1.59 (1.72)

*SD* Standard Deviation

Most considered themselves heterosexual (96.5 %), 2.2 % bisexual, and 1.1 homosexual. The mean age for first vaginal intercourse was 14.57 (*SD* = 1.29), and 51 % reported using condoms consistently in their sexual relationships.

#### Assessment of the models

First, we assessed whether the TPB, SCM, and IMB model would fit our data using structural equation modeling. The adjustment of each model is shown in Table 2. Second, we analyzed the bivariate correlations between the estimated constructs.

**Table 2:** Summary of goodness of fit of models

Theoretical model	Chi-squared test				Indices		
	χ^2^	*df*	χ^2^/*df*	*p*	CFI	TLI	RMSEA
TPB	13.56	8	1.69	≤0.001	0.98	0.97	0.045
SCM	57.47	26	2.21	≤0.001	0.93	0.88	0.061
IMB	196.09	48	4.08	≤0.001	0.98	0.97	0.010

*TPB* Theory of Planned Behavior, *SCM* Socio-Cognitive Model, *IMB* Information-Motivation-Behavioral Skills Model, *χ2* Chi-square, *df* degrees of freedom, *p*, p-value, *CFI* Comparative Fit Index, *TLI* Turker-Lewis Index, *RMSEA* Root Mean Square of Approximation

#### Theory of planned behavior

**Fig 1:**
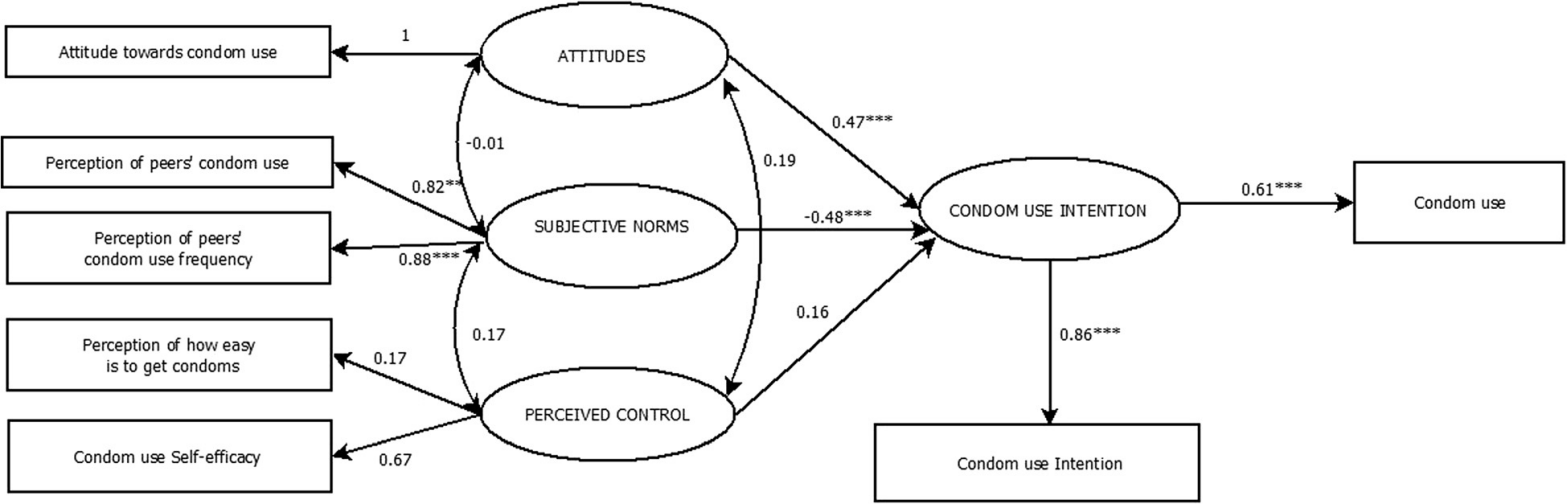
Theory of Planned Behavior Model predicting condom use. Significant paths are indicated by asterisks: *p < 0.05, **p < 0.01, ***p < 0.001

The TPB showed an adequate fit (χ^2^ (*n* = 410, 8) = 13.56; χ^2^/df = 1.69), with indices close to 1 (CFI = 0.98, TLI = 0.97) and a value of RMSEA lower than 0.06 (RMSEA = 0.045, 90 % CI [0.001, 0.085]). The TPB standardized coefficients are shown in Fig. 1.

#### Socio-cognitive model

**Fig 2:**
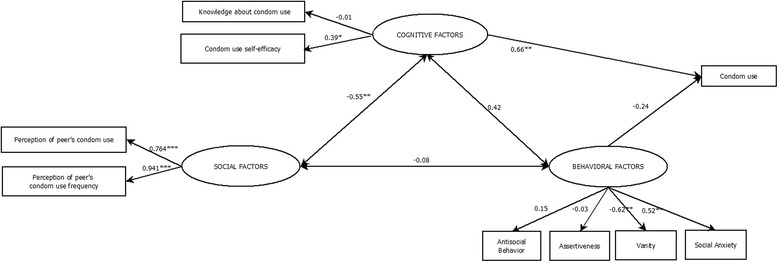
Socio-Cognitive Model predicting condom use. Significant paths are indicated by asterisks: *p < 0.05, **p < 0.01, ***p < 0.001

The adjustment indices of the SCM indicate a poor fit with indices lower than 0.95 (CFI = 0.93, TLI = 0.88), although an acceptable RMSEA value can be lower than 0.080 (RMSEA = 0.061, 90 % CI [0.039, 0.082]). Figure 2 shows the SCM and its standardized coefficients.

#### Information-motivation-behavioral skills model

**Fig 3:**
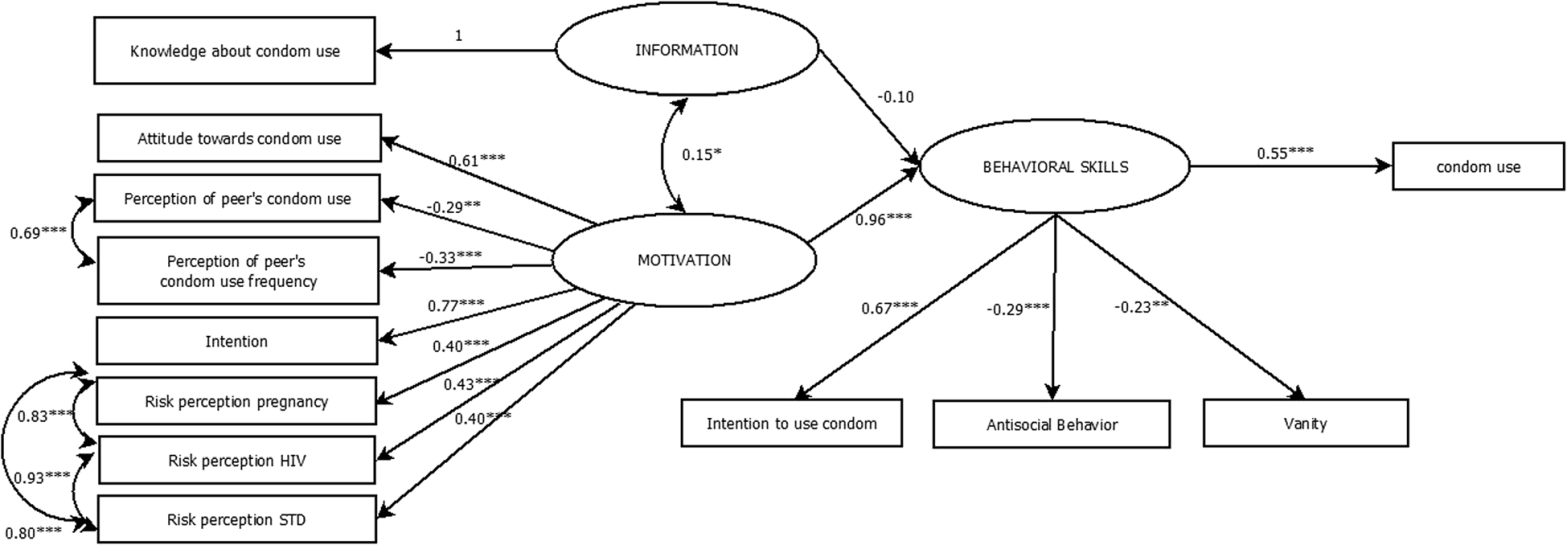
Information-Motivation-Behavioral Skills Model predicting condom use. Significant paths are indicated by asterisks: *p < 0.05, **p < 0.01, ***p < 0.001

The IMB model shows an adequate fit with CFI and TLI indices close to 1 (CFI = 0.98 and TLI = 0.97). However, the RMSEA value (error) was higher than 0.08 (RMSEA = 0.10; 90 % CI [0.083–0.112]), and χ^2^/df value was higher than 3 (χ^2^ (*n* = 410, 48) = 196.09) indicating a poor adjustment for this model. Figure 3 shows the IMB model and its standardized coefficients.

### Bivariate correlations between the estimated factors

#### Theory of planned behavior

The results showed that having a favorable attitude toward condom use (β = 0.47, *p* < 0.001) and perceiving that peers use condoms in their sexual relationships (β = 0.48, *p* < 0.001) were directly related to the intention to use condoms. Interestingly, condom use self-efficacy was not directly related to behavior, nor indirectly through condom use intention. Condom use intention was a significant predictor of the FCU in adolescents (β = 0.61, *p* < 0.001).

#### Socio-cognitive model

The SCM comprises cognitive, behavioral, and social components. The cognitive component was measured using knowledge about STIs and condom use and condom use self-efficacy. The behavioral component was determined using measures of several skills that may be related to condom use, such as assertiveness, naturalness and humility (vanity), antisocial behavior, and ability to manage social situations without anxiety (social anxiety). Finally, the social component comprised measures of subjective norms related to condom use. Compared to the others, the cognitive component was more related to the FCU in adolescents (β = 0.66, *p* < 0.01).

#### Information-motivation-behavioral skills model

The IMB model comprises three components: information, motivation, and behavioral skills. The motivational component included attitudes toward condom use, subjective norms, condom use intention, and sexual risk perception toward pregnancy, HIV, and other STIs. This component was significantly related to behavioral skills (β = 0.96, *p* < 0.001), but the information component was not. The behavioral skills component was related to the FCU in adolescents. The information and motivation components were positively associated, which indicates that adolescents with a higher level of knowledge about STIs and condom use are more likely to be motivated to protect themselves from STIs and unplanned pregnancies via condom use. The behavioral skills component was positively and significantly related to condom use according to the IMB model.

#### Comparison between theoretical approaches

According to the goodness-of-fit indices, the TPB and IMB model had the highest values for CFI and TLI (≥0.97), while SCM showed the lowest indices (CFI = 0.93; TLI = 0.88). Compared to the TPB, the IMB model had the highest value of error (RMSEA = 0.10), and provided the highest chi-square value (χ^2^ (*n* = 410, 48) = 196.09). The TPB and SCM had acceptable values of error (RMSEA ≤ 0.08) compared to the IMB model (0.10).

## Discussion

In this study, three socio-cognitive models for predicting the FCU in Spanish adolescents were evaluated. The results revealed that the TPB best contributed to the prediction of the FCU in this study, at least when compared with the other two models. Furthermore, condom use intention, motivational and cognitive-attitudinal factors (i.e., HIV and STI knowledge, attitude towards condom use, subjective norms), and behavioral skills were involved in predicting FCU in Spanish adolescents.

The three models are comprised of similar constructs, although they offer a different approach to predict health behaviors, including condom use. Compared to the TPB, the SCM and IMB model consider knowledge as a cognitive factor. The SCM has a specific behavioral component, including skills to succeed in a particular situation, such as condom use negotiation. The IMB model also includes the behavioral skills component as a direct predictor of the behavior. It is important to note that the premise that skills, abilities, and environment constructs can moderate the relationship between intention-behavior was not included in the original TPB, yet it is recognized in the lasting formulation of a reasoned action approach, which is the integrative model of behavioral prediction [52, 53].

In the analysis of the contribution of each construct for the prediction of the FCU, intention in the TPB proved to be good predictors of the behavior. The correlation between condom use intention and condom use behavior was moderate (0.61). This is higher than values in previous studies: Albarracín et al. [15] recorded 0.57 and Sheppard et al. [20] 0.53; however, this was slightly lower than Van den Putte et al. at 0.62. Several studies indicate that the association between condom use intention and the use of this method of protection tends to be higher when past condom use is evaluated, rather than when a prospective measure is evaluated [15]. This happens because the intention to engage in a behavior is closely related to past behaviors [54]. Based on this premise, the intention-behavior correlations may be overestimated in this study since a retrospective measure of condom use was undertaken.

Compared to subjective norms and perceived control, attitude toward condom use was the best predictor of intention to use condoms. This result indicates that the FCU in Spanish adolescents mainly depends on their attitude toward this method of protection, rather than their peers’ FCU or self-efficacy to manage obstacles in using this method. Meta-analytic studies agree with our study on the importance of attitude compared to subjective norms in the prediction of condom use [14, 19].

In this study, self-efficacy and perceived control were interchangeable constructs [5, 52]. In the estimated model for the TPB, the correlation between perceived control/self-efficacy and condom use intention was non-statistically significant, suggesting that a higher perceived/self-efficacy control is not associated with condom use intention. In a critical review of 11 peer-reviewed studies, Protogerou et al. [55] found a small correlation (0.04) between perceived control and condom use in the prediction of condom use in South African university students. Consistently, the meta-analysis conducted by Albarracín and colleagues [15] concluded that perceived behavioral control does not contribute significantly to condom use, which is in contrast to the TPB. These unexpected results can be explained by our use of a brief self-efficacy scale focus on skills to manage obstacles to use condom. Another possible explanation is that self-efficacy and perceived control constructs are not equivalent [10, 56]. This study demonstrates that the perception of being an effective person to manage condom use obstacles does not contribute to the prediction of condom use. It is important to note that although specific self-efficacy does not contribute to the prediction of condom use in this study, some evidence exists of its mediation role on the efficacy of sexual-risk reduction intervention for adolescents [41, 57, 58], and prediction of condom use indirectly via intention [37].

Cognitive factors, including knowledge and self-efficacy, contributed to the explanation of the FCU to a greater extent compared to behavioral factors in the SCM. While the importance of having the skills to use condoms and condom negotiation with one’s partner is recognized, it appears that self-efficacy to manage obstacles for condom use is a key predictor of condom use frequency. Notably, knowledge about STIs and condom use had the lowest standardized weight in the SCM (−0.01) and the IMB model (0.10), and were non-significant in the paths. This result does not empirically support the hypothesis that young people with more knowledge about STIs and condoms undertake greater condom use. However, this is not a definite conclusion—many factors could have contributed to this low explanatory contribution; for example, the instruments and scales we used could have been too broad in focus, meaning that they did not properly evaluate specific knowledge about condom use [16]. Therefore, we recalculated the SCM and IMB model using only the subscale “Knowledge about condom use” as a measure of knowledge. However, the goodness of fit for both models worsened significantly, possibly due to the small number of items that comprise this factor. Testing the applicability of the IMB model, knowledge about HIV has been shown to be influential in the prediction of condom use among students from South Africa [37]; however, most studies have suggested that knowledge is not a significant direct determinant of the behavior [35, 59].

### Limitations

Among this study’s limitations is the use of self-reports to measure behavioral variables. Cross-sectional data is not ideal for testing models since the intention to engage in a behavior is closely related to past behaviors [38, 43]; nonetheless, it is still suitable for this purpose [60]. It would have been more appropriate using a more specific measure of condom use rather than frequency of condom use, for example, condom use at the last vaginal sex. Theoretical models are usually tested with self-report measures despite evidence of greater social desirability regarding more objective measures [19]. The use of biological measures for STIs is not feasible when most participants are not sexually experienced [41]. Although the sample is geographically dispersed, a non-representative sample was selected and as they are from a developed country, caution should be exercised regarding interpretation of the generalizability of the results. When using a 4-point scale (rather than a 5-point scale), the conceptual distances between the response options are no longer “relatively equal” and non-parametric tests are recommended. In the present study, we selected the robust Weighted Least Squares Mean and Variance (WLSMV) method because it is suitable and recommended for categorical measures [49]. Accurate measurement of constructs is a challenge when comparing several theoretical models [39]. In the present study, “perceived norms” focused on descriptive norms. Future studies should also include injuctive norms, which refer to adolescents’ perceptions of what their significant referents think about performing a certain behavior. Compared to other health behaviors, such as physical activity and dietary behaviors, the relationship between perceived control and sexual behavior is much lower [18]. The classic meta-analytic review of the efficacy of the TPB conducted by Armitage et al. [19] concluded that perceived control correlates strongly to condom use intention rather than behavior, and value for perceived control over behavior was .003 %. More evidence is needed on perceived control as a predictor of behavior. Based on previous studies [41], it must be noted that a “reasoned” approach can be not the most appropriate to predict condom use due to the emotional’ nature of the behavior and the circumstances it occurs in.

## Conclusions

This study provided new empirical evidence of the plausibility of the TPB, SCM, and IMB model in predicting the FCU among adolescents. Based on the data, the TPB was the most suitable model compared to the other models. Further research is required to confirm these results, particularly using a more complete measure of self-efficacy. We also found that the FCU for adolescents in Spain can be predicted by their intention to use condoms. The promotion of sexual health can benefit from our results via intervention strategies that aim to improve attitudes toward condom use and encourage adolescents to view condom use as more acceptable via peer condom use. Both objectives contribute directly to the prediction of condom use intention, which is a direct determinant of the FCU. In general, socio-cognitive models, particularly the TPB, are useful for understanding Spanish adolescents’ sexual risk and to positively contribute to the design of preventive interventions for HIV, other STIs, and unwanted pregnancies. As condom use intention is strongly associated with condom use, preventive programs should focus on improving attitudes toward condom use and the normative perception regarding peers’ condom, as both are direct precursors of intention to use condoms. Therefore, increasing condom use intention means that the probability of using condoms is higher. The limitations of the present study must be addressed in future research to obtain results that are more generalizable to adolescents. In order to obtain a more comprehensive understanding of health behaviors [43], longitudinal designs are needed that test the accuracy of the socio-cognitive models in multiple samples, as well as the use of different dependent variables, so that evidence of the generalizability of health behavior theories across behaviors is obtained. Socio-cognitive models contribute positively to the prediction of condom use. In order to improve the ability to predict condom use in adolescents, the next step should be testing integrative models that include personal, contextual, environmental, and social factors. Future studies should test multi-level frameworks that provide a broader understanding of sexual behaviors in adolescents.

## Additional file

Additional file 1:
**The English version of the measures used for the present study.** (DOCX 36 kb)

## Abbreviations

TPB: Theory of planned behavior
SCM: Socio-cognitive model
IMB: Information-motivation-behavioral skills
FCU: Frequency of condom use
HIV: Human immunodeficiency virus
STIs: Sexually transmitted infections
WLSMV: Weighted Least Squares Mean and Variance
CFI: Comparative Fit Index
TLI: Tucker-Lewis Index
RMSEA: Root Mean Square Error of Approximation
