# Supplementary material for: Predicting condom use in adolescents: a test of three socio-cognitive models using a structural equation modeling approach
Source: BMC Public Health. 2016 Jan 14;16:35. doi: 10.1186/s12889-016-2702-0 (PMC4712548; doi:10.1186/s12889-016-2702-0)
Supplement: Additional file 1: — The English version of the measures used for the present study. (DOCX 36 kb) [file 12889_2016_2702_MOESM1_ESM.docx]

**Gender: □** Male **□** Female

**Socioeconomic level:**

¿How many computers are there where you live?

**□** 1 **□** 2 **□** 3 **□** 4  **□** 5 or more

¿How many televisions are there where you live?

**□** 1 **□** 2 **□** 3 **□** 4  **□** 5 or more

¿How many room are there where you live?

**□** 1 **□** 2 **□** 3 **□** 4  **□** 5 **□** 6 **□** 7 or more

* Kitchen, bathrooms, terraces, living rooms, dining are not included.

**Age: _____**

**Date of birth: _________________**

**Province: _____________________**

**City: ________________________**

**High school: _____________________________**

**Level: ________________________**

**Family situation:**

**□** Married parents

**□** Separate or divorce parents

□ Single parents

□ Orphan

□ Other

1. **Read each of the following statements and select the most appropriate for you. Remember: There is no right or wrong answers.**

**Strongly Strongly**

**Disagree Disagree Agree Agree**

1. Using a condom during sex consistently is good to prevent STI transmission

and unplanned pregnancies …………………………………………………………………………………………

1. Carrying condoms for my use makes easy to use them in a sexual relationship..…………
2. Confessing to your friends adolescents that you use a condom during sex is useful to

promote condom use among them………….…………………………………………………………..........

1. Defending the use of condom publicly is good to promote condom use during sex……...
2. **Read each of the following statements and select the most appropriate for you. Remember: There are no right or wrong answers.**

**Strongly Strongly**

**Disagree Disagree Agree Agree**

1. If my partner would ask me to have unprotected sex I would refuse it. ………………………….…
2. If my partner would want to have sex without a condom, I would try to

convince her/him to use it…………………………………………………………………………………………….……

1. If I were going to have sex and realize that I have no condoms, I would wait until

I have them to maintain sexual intercourse.………….………………….......………………………………….

|  | **Not sure** | **Probably not** | **Maybe** | **Probably yes** | **Sure** |
| --- | --- | --- | --- | --- | --- |
| **C) In the next 12 months, how likely is you will use a condom if I have a sexual relationship** | (1) | (2) | (3) | (4) | (5) |

| 1. **Have you ever had…** | | **How old were you at the first time…?** |
| --- | --- | --- |
| Petting | □ YES □ NO |  |
| Vaginal intercourse | □ YES □ NO |  |
| Anal sex | □ YES □ NO |  |
| Oral sex | □ YES □ NO |  |
| Mutual masturbation | □ YES □ NO |  |

**E) Do you use any of these methods of protection in your sexual relationships?**

□ No method of prevention of diseases or pregnancy

□ Condom. If you have checked this option, please, indicate what percentage of times you use this method of protection in

      your sexual relationships (with a circle around the approximate percentage):

|  |  |  |  |  |  |  |  |  |  |  |  |  |  |  |  |  |  |  |  |
| --- | --- | --- | --- | --- | --- | --- | --- | --- | --- | --- | --- | --- | --- | --- | --- | --- | --- | --- | --- |
|  |  |  |  |  |  |  |  |  |  |  |  |  |  |  |  |  |  |  |  |

0% 5% 10% 15% 20% 25% 30% 35% 40% 45% 50% 55% 60% 65% 70% 75% 80% 85% 90% 95% 100%

□ **Anticonceptive pill.** If you have checked this option, please, indicate what percentage of times you use this method of protection in

      your sexual relationships (with a circle around the approximate percentage):

|  |  |  |  |  |  |  |  |  |  |  |  |  |  |  |  |  |  |  |  |
| --- | --- | --- | --- | --- | --- | --- | --- | --- | --- | --- | --- | --- | --- | --- | --- | --- | --- | --- | --- |
|  |  |  |  |  |  |  |  |  |  |  |  |  |  |  |  |  |  |  |  |

0% 5% 10% 15% 20% 25% 30% 35% 40% 45% 50% 55% 60% 65% 70% 75% 80% 85% 90% 95% 100%

□ **Other methods** (Please, indicate which ones): __________________________________________________________________

**F) You consider yourself:** □ Heterosexual □ Bisexual □ Homosexual

**G) How many sexual partners with penetration have you had in the last six months? _____**

1. **Do you think that your same-age friends use condoms in their sexual relationships? □** YES □ NO

**I) How often do you think your same-age friends use a condom in their sexual relationships?**

□ Always □ Most of the times □ Sometimes □ Never

1. **Do you think that it is easy to get condoms?**  □ YES □ NO

**K) How much risk you think there is in the following actions?**

|  | *Risk of…* | | |
| --- | --- | --- | --- |
|  | *Unwanted pregnancy* | *HIV transmission* | *STIs transmission* |
| 1. Having oral sex without condom | □ no risk at all □ just a little  □ quite a bit □ a lot | □ no risk at all □ just a little  □ quite a bit □ a lot | □ no risk at all □ just a little  □ quite a bit □ a lot |
| 2. Having vaginal sex without condom | □ no risk at all □ just a little  □ quite a bit □ a lot | □ no risk at all □ just a little  □ quite a bit □ a lot | □ no risk at all □ just a little  □ quite a bit □ a lot |
